# Annual Reports of the Commissioners and Superintendent of the Indiana Hospital for the Insane

**Published:** 1855-03

**Authors:** 


*Annual Reports of the Commissioners and Superintendent of the Indiana Hospital for the Insane.*

An interesting and well digested document.

